# OXTR rs2254298 polymorphism influences escitalopram response in Generalized Anxiety Disorder: a sex-specific role for oxytocin signaling

**DOI:** 10.3389/fpsyt.2025.1718106

**Published:** 2025-11-21

**Authors:** Liang Xue, Hongfen Ni, Hong Dai

**Affiliations:** 1 Department of Psychiatry, Huzhou Third Municipal Hospital, the Affiliated Hospital of Huzhou University, Huzhou, China; 2 Department of Laboratory Medicine, Huzhou Third Municipal Hospital, the Affiliated Hospital of Huzhou University, Huzhou, China

**Keywords:** Generalized Anxiety Disorder, oxytocin, OXTR gene, pharmacogenetics, sex differences, treatment response, SSRI

## Abstract

**Objective:**

This study aimed to investigate whether the functional OXTR rs2254298 polymorphism moderates escitalopram response in Generalized Anxiety Disorder (GAD) through oxytocin (OT) neuroendocrine signaling, with specific consideration of sex-specific effects.

**Methods:**

88 patients with GAD and 92 matched healthy controls (HCs) were enrolled. All participants underwent OXTR rs2254298 genotyping and baseline serum OT measurement. GAD patients then received 8 weeks of escitalopram monotherapy. Anxiety severity was assessed using the Hamilton Anxiety Scale (HAMA) at baseline and weeks 2, 4, and 8.

**Results:**

Baseline serum OT levels were significantly elevated in GAD patients compared to HCs (126.28 ± 88.41 vs. 92.77 ± 47.51 pg/mL, p = 0.006), a difference that was primarily driven by female patients (*p* = 0.037). OT levels were positively correlated with baseline HAMA scores (*r* = 0.197, *p* = 0.008). After 8 weeks of treatment, the OXTR rs2254298 genotype distribution significantly differed between treatment responders and non-responders (*p* = 0.049), with GG homozygotes showing a lower response rate.

**Conclusion:**

Our findings suggest that the OXTR rs2254298 polymorphism may influence escitalopram response in GAD via OT neuroendocrine mechanisms, exhibiting prominent sexual dimorphism. Integrating genetic profiling with endocrine biomarkers holds promise for personalizing anxiety treatment.

## 1 Introduction

Generalized Anxiety Disorder (GAD) is a prevalent and disabling condition characterized by persistent, excessive worry leading to significant functional impairment (1). Selective serotonin reuptake inhibitors (SSRIs), such as escitalopram, are first-line treatments due to their established efficacy and safety profile (2, 3). Nonetheless, a substantial portion of patients (30-40%) exhibit poor or delayed treatment response (4). This interindividual heterogeneity underscores an urgent need to identify predictive biomarkers and elucidate the underlying biological mechanisms to advance personalized medicine in psychiatry.

Beyond the serotonergic system, the neuropeptide oxytocin (OT) has garnered significant interest for its critical role in modulating stress responses, social cognition, and emotional regulation—processes profoundly altered in GAD (5). The OT system exhibits a complex, sometimes paradoxical, relationship with anxiety. While renowned for its anxiolytic and pro-social properties (5), clinical studies have consistently reported elevated peripheral OT levels in anxiety disorders (6). This has led to the ‘compensatory hypothesis’, suggesting that chronic anxiety may trigger an adaptive upregulation of OT secretion to counteract distress; however, chronic elevation might eventually lead to receptor downregulation or functional desensitization, potentially exacerbating the pathological state (6, 7).

The functionality of the OT system is significantly influenced by genetic variation. A single-nucleotide polymorphism (rs2254298, A>G) in the oxytocin receptor gene (OXTR) has been extensively associated with altered social stress reactivity and susceptibility to anxiety and depression (8, 9). Notably, the effects of this polymorphism are often moderated by environmental factors and exhibit sex-specific patterns (10), highlighting complex gene-environment and gene-sex interactions. Moreover, recent studies have highlighted that OXTR polymorphisms interact with early environmental factors such as parenting experiences to shape social and emotional outcomes (11), underscoring the importance of gene-environment interplay in anxiety-related traits. Recent independent studies have replicated the association between the OXTR rs2254298 GG genotype and increased risk for panic spectrum symptoms (12), alcohol-withdrawal anxiety (13), and childhood anxiety problems (14), underscoring a cross-disorder vulnerability profile that our pharmacogenetic data now extend to SSRI response.

Despite this growing body of evidence, a critical gap remains. Previous studies have often examined these facets—peripheral OT levels, OXTR genetics, and clinical anxiety—in isolation. It remains unexplored whether genetic variation in the OXTR gene influences response to first-line pharmacological treatments like SSRIs, and whether this relationship is mediated by underlying alterations in oxytocin neuroendocrine signaling and is modulated by sex. Given the intricate interplay between the serotonergic and oxytocinergic systems in stress-related circuits, we hypothesized that the OXTR rs2254298 genotype moderates escitalopram treatment response in GAD, potentially through a mechanism involving OT neuroendocrine signaling and exhibiting sex-specific effects.

This study therefore employed a multidisciplinary approach to concurrently investigate: (1) baseline serum OT levels between GAD patients and healthy controls (HCs), with consideration of sex differences; (2) the association between OXTR rs2254298 genotype and escitalopram treatment response; and (3) the interrelationships between OT levels, genotype, clinical severity, and treatment outcome. We hypothesized that 1) GAD patients would exhibit dysregulated OT levels compared to HCs, with sex differences; 2) the OXTR rs2254298 genotype would be associated with differential response to escitalopram; and 3) these genetic and endocrine factors would be intercorrelated.

## 2 Materials and methods

### 2.1 Participants

The study was approved by the Ethics Committee of Huzhou Third Municipal Hospital (Approval No: 2020-116). Based on previous studies investigating genotype-phenotype associations in psychiatric pharmacogenetics with medium effect sizes (f ≈ 0.25-0.3), *a priori* power analysis using G*Power software indicated that a sample size of 80 patients would provide 80% power (α = 0.05) to detect such effects. Eighty-eight patients meeting ICD-10 criteria for GAD were recruited from the outpatient clinic or inpatient department of Huzhou Third People’s Hospital between August 2021 and June 2023. Inclusion criteria were: (1) age 18–65 years; (2) HAMA score ≥14 and HAMD-17 score <17; (3) either medication-naïve or medication-free for at least 2 weeks prior to enrollment; (4) provision of written informed consent.

Ninety-two gender- and age-matched healthy controls (HCs) were recruited from hospital staff and community volunteers. HCs had HAMA scores <7 and no history of psychiatric disorders.

Common exclusion criteria for all participants included: (1) comorbid Axis I psychiatric disorders; (2) history of substance abuse; (3) significant physical or chronic illness.

The final sample consisted of 88 GAD patients (64 females, mean age 50.81 ± 11.21; 24 males, mean age 49.33 ± 12.56) and 92 HCs (51 females, 41 males). The female-to-male ratio (~3:1) reflects the higher prevalence of GAD in women. There were no significant age differences between groups (*p* > 0.05). This study was conducted in accordance with the Declaration of Helsinki and was approved by the Ethics Committee of Huzhou Third Municipal Hospital (Approval No: 2020-116). All participants provided written informed consent.

### 2.2 Treatment and assessment

Patients received a fixed regimen of escitalopram (initially 10 mg/day, potentially increased to 20 mg/day after one week) for 8 weeks. No other psychotropics were allowed except for zolpidem (10 mg) for severe insomnia. Anxiety severity was assessed using the HAMA at baseline, week 2, week 4, and week 8. Treatment response was defined as *a* ≥50% reduction in HAMA total score from baseline.

### 2.3 Biochemical and genetic analyses

Fasting venous blood was collected between 8:00 and 10:00 AM. Serum OT levels were measured using a commercial ELISA kit (Wuhan Saipai Biotechnology Co., Ltd., China). The intra- and inter-assay coefficients of variation were <10% and <15%, respectively. Genomic DNA was extracted from whole blood. The OXTR rs2254298 polymorphism was genotyped using TaqMan-based real-time PCR on a Bio-Rad CFX96 system. Genotyping accuracy was confirmed by repeating 10% of randomly selected samples, with 100% concordance. All laboratory personnel were blinded to clinical status. These assays were conducted to identify potential biomarkers predictive of treatment response, with the goal of advancing personalized medicine in psychiatry.

### 2.4 Statistical analysis

Data were analyzed using SPSS 20.0. Normality was assessed using the Shapiro-Wilk test. Between-group comparisons of OT levels (log-transformed for normality) were performed using t-tests or ANOVA. Categorical data were compared using the χ² test. Correlations were assessed using Spearman’s rank correlation. Hardy-Weinberg equilibrium (HWE) was tested for the genotype distribution. A multivariate logistic regression model was employed to identify predictors of treatment response (dependent variable: response at week 8; independent variables: sex, age, log(OT), and genotype [additive model]). A two-tailed p-value < 0.05 was considered statistically significant. The absence of multicollinearity among independent variables was confirmed by variance inflation factors (VIF) all being below 2.0.Given the exploratory nature of some analyses and the multiple comparisons conducted, findings should be interpreted with caution. The primary focus was on *a priori* hypotheses, which are less susceptible to inflation of Type I error. All statistical analyses were performed under the guidance of a qualified biomedical statistician (H.N.) to ensure the validity and appropriateness of the methods.

## 3 Results

### 3.1 Oxytocin levels and clinical correlations

Baseline serum OT levels were significantly higher in the GAD group (126.28 ± 88.41 pg/mL) compared to HCs (92.77 ± 47.51 pg/mL) (*t* = -3.12, p = 0.006). This elevation was driven primarily by female patients (GAD females: 138.02 ± 94.65 pg/mL vs. HC females: 101.11 ± 48.90 pg/mL; *p* = 0.037). No significant difference was found between male groups (*p* = 0.447). A significant positive correlation was observed between baseline OT levels and HAMA scores in the patient group (*r* = 0.197, *p* = 0.008).

### 3.2 Genetic analyses

The genotype distributions for the OXTR rs2254298 polymorphism were in Hardy-Weinberg equilibrium in both case and control groups (*p* > 0.05). There were no significant differences in allele or genotype frequencies between GAD patients and HCs (**Table 1**).

**Table 1:** Distribution of OXTR rs2254298 genotype and allele frequencies in GAD patients and healthy controls.

Group	n	AA n(%)	AG n(%)	GG n(%)	A allele n(%)	G allele n(%)
GAD	88	13(14.8)	43(48.9)	32(36.4)	69(39.2)	107 (60.8)
Controls	92	17(18.5)	31(33.7)	44(47.8)	65(35.3)	119(64.7)
χ^2^				4.287	0.579	
p				0.117	0.447	

### 3.3 Genotype and treatment response

Response rates increased to 82% by week 8. A significant association was found between OXTR rs2254298 genotype and treatment response (χ² = 6.021, p = 0.049,Cramer’s V = 0.262), with GG homozygotes exhibiting a significantly lower response rate compared to A-allele carriers (**Table 2**).

**Table 2:** Association between OXTR rs2254298 genotype and escitalopram response in GAD patients at week 8.

Genotype	Responders (n = 72)	Non-responders(n = 16)	P
AA	11*(*15.3%*)*	2 *(*12.5%*)*	0.049
AG	39 *(*54.2%*)*	4 *(*25.0%*)*	
GG	22*(*30.6%*)*	10*(*62.5%*)*	

### 3.4 Multivariate logistic regression

In the logistic regression model adjusted for sex, age, and log-transformed OT level, the OXTR rs2254298 genotype was not an independent predictor of treatment response at week 8 (OR = 1.760, 95% CI: 0.601–5.152, *p* = 0.302) (**Table 3**).

**Table 3:** Multivariate logistic regression for predictors of treatment response in GAD patients at week 8.

Variable	B	SE	Wald	p	OR(95% CI)
Sex(Female)	-1.256	1.125	1.247	0.264	0.285(0.031-2.581)
Age	0.020	0.033	0.367	0.544	1.020(0.957-1.087*)*
Log(Oxytocin)	0.877	1.294	0.460	0.498	2.403(0.190-30.332*)*
rs2254298 Genotype*	0.565	0.548	1.064	0.302	1.760(0.601-5.152*)*

*Genotype coded additively (number of A alleles).

## 4 Discussion

### 4.1 Principal findings

To our knowledge, this is the first study to integrate OXTR genotyping, peripheral OT level assessment, and longitudinal SSRI treatment response evaluation in patients with GAD, revealing a sexually dimorphic pattern. Our findings provide novel evidence that the oxytocin system, modulated by genetic susceptibility and sex differences, may play an important moderating role in the pharmacological treatment of GAD. We report three principal findings: (1) patients with GAD exhibited significantly elevated baseline serum OT levels compared to healthy controls, a difference driven primarily by female patients;(2) OT levels were positively correlated with baseline anxiety severity; and (3) the OXTR rs2254298 genotype was associated with differential treatment response at week 8.

### 4.2 Oxytocin dysregulation in GAD and sex-specific effects

The elevated OT levels observed in GAD patients align with the *“*compensatory hypothesis*”* of anxiety disorders (7). The significant correlation between OT levels and HAMA scores further suggests that the degree of OT dysregulation may reflect clinical severity. A notable and novel finding of our study is the sex-specific effect in OT elevation, which was significant only in female patients. This observed sexual dimorphism could be attributable to estrogen*’*s known role in upregulating OXTR expression and enhancing OT signaling (15), though this remains a hypothetical mechanism as estrogen levels were not directly measured in our study. These results underscore the necessity of considering sex as a critical biological variable in psychoneuroendocrine research.

### 4.3 OXTR genotype as a moderator of treatment response

A key and intriguing finding is the time-dependent association between OXTR rs2254298 and treatment response, which emerged only at week 8. Although the genotype was not an independent predictor in the multivariate model adjusting for OT levels, this pattern of results is highly suggestive of a more complex relationship rather than a null effect. We hypothesize that OT may mediate the effect of OXTR genotype on treatment response.

(16), though this requires formal mediation analysis in future studies. Our results suggest an association where the OXTR rs2254298 variant may influence escitalopram response through a neuroendocrine mechanism involving OT signaling, particularly in women. Future studies should also consider epistatic interactions between OXTR and other neurotransmitter-related genes, such as those in the dopaminergic system, which have been shown to influence social and emotional processes (17). This sexually dimorphic effect may be driven by estrogen-mediated OXTR expression, highlighting the importance of considering sex as a biological variable in psychopharmacology. This leads to the consideration of a more integrated model linking genetics, neuroendocrinology, and clinical outcome. It is crucial to note that these findings are correlational, and future experimental studies are required to establish causality. Beyond anxiety-specific mechanisms, the association of the OXTR rs2254298 with broader physiological and psychological processes, such as sleep latency and personal distress (18), suggests that this polymorphism may influence escitalopram response by modulating fundamental systems of arousal and emotional regulation.

### 4.4 An integrated neuroendocrine model and clinical implications

Although the genotype was not an independent predictor in the multivariate model, the observed pattern supports a potential mediating role of OT levels between genetics and treatment outcome. Future studies with longitudinal OT sampling and larger samples are needed to formally test this mediation model. Our study supports the potential for integrating genetic and endocrine biomarkers to personalize anxiety treatment. Our findings align with emerging evidence that genetic variations in both the oxytocinergic and serotonergic systems modulate clinical outcomes in stress-related disorders (19). Specifically, women with GAD and certain OXTR genotypes may represent a subgroup that exhibit distinct OT profiles and differential SSRI responses. For example, our data raise the possibility that women with GAD and the GG genotype might be candidates for earlier consideration of alternative therapies (e.g., SNRIs or psychotherapy), or OT-augmentation strategies. However, these are speculative suggestions that require validation in prospective clinical trials.

### 4.5 Limitations and future directions

This study has several limitations. First, although our sample size was based on an *a priori* power calculation and is comparable to many preliminary pharmacogenetic studies, it remains modest. This limits the generalizability of our findings and the power to detect smaller effect sizes, particularly in subgroup analyses (e.g., within male participants alone). Future studies with larger cohorts are needed to confirm and extend our observations. Second, single-timepoint OT measurement may not capture dynamic changes. Third, future studies should account for environmental factors such as childhood trauma, which may interact with OXTR genetics. Epigenetic studies have shown that OXTR variations, in conjunction with early adverse experiences like child abuse, significantly influence adult psychiatric symptom profiles (20), highlighting the need for integrated gene-environment models in GAD research. For example, a recent study in schizophrenia demonstrated that OXTR polymorphisms moderated the impact of childhood trauma on social functioning (21). Incorporating such measures will provide a more comprehensive etiological model of GAD and its treatment.

Despite these limitations, our study demonstrates the value of an integrated, multi-level approach in psychoneuroendocrinology. By combining genetic and endocrine biomarkers, we highlight the potential to identify subgroups of GAD patients—particularly women—who may exhibit a distinct neuroendocrine profile and differential response to SSRIs. These findings encourage a shift from a one-size-fits-all treatment model toward biomarker-guided personalization in psychiatry. Future research should focus on formally testing the proposed mediation models and exploring the utility of OT-based augmentation strategies for SSRI non-responders.

## Data Availability

The original contributions presented in the study are publicly available. This data can be found here: Figshare repository, DOI: 10.6084/m9.figshare.30272095.
