# Correction: OXTR rs2254298 polymorphism influences escitalopram response in Generalized Anxiety Disorder: a sex-specific role for oxytocin signaling

**DOI:** 10.3389/fpsyt.2025.1754488

**Published:** 2025-12-10

**Authors:** Liang Xue, Hongfen Ni, Hong Dai

**Affiliations:** 1 Department of Psychiatry, Huzhou Third Municipal Hospital, the Affiliated Hospital of Huzhou University, Huzhou, China; 2 Department of Laboratory Medicine, Huzhou Third Municipal Hospital, the Affiliated Hospital of Huzhou University, Huzhou, China

**Keywords:** Generalized Anxiety Disorder, oxytocin, OXTR gene, pharmacogenetics, sex differences, treatment response, SSRI

The author affiliations in the published paper were erroneously given as:

“Liang Xue^1,2^, Hongfen Ni^2,3^, Hong Dai^1,2^

^1^Department of Psychiatry, Huzhou Third Municipal Hospital, Huzhou, China

^2^The Affiliated Hospital of Huzhou Normal College, Huzhou, China

^3^Department of Laboratory Medicine, Huzhou Third Municipal Hospital, Huzhou, China”

The correct author list and affiliations read:

“Liang Xue^1*^, Hongfen Ni^2^, Hong Dai^1^

^1^Department of Psychiatry, Huzhou Third Municipal Hospital, the Affiliated Hospital of Huzhou University, Huzhou, China

^2^Department of Laboratory Medicine, Huzhou Third Municipal Hospital, the Affiliated Hospital of Huzhou University, Huzhou, China”

The original version of this article has been updated.

